# Risk Stratification for Early Seizures after Traumatic Brain Injury: A Clinical Scoring Model for Prediction and Management

**DOI:** 10.1177/2689288X251377020

**Published:** 2025-09-23

**Authors:** Hidetaka Onda, Mizuki Kojima, Nodoka Miyake, Kenta Shigeta, Naoki Tominaga, Shoji Yokobori

**Affiliations:** ^1^ Department of Disaster and Emergency Medicine, Kochi University, Kochi, Japan.; ^2^ Department of Emergency and Critical Care Center, Nippon Medical School Hospital, Tokyo, Japan.

**Keywords:** acute subdural hematoma, levetiracetam, post-traumatic epilepsy, prophylactic therapy

## Abstract

Early seizures within 1 week after traumatic brain injury (TBI) are classified as acute symptomatic seizures, which can worsen secondary brain injury, leading to poor neurological outcomes and increased mortality. Despite prophylactic antiepileptic drug (AED) use, early seizures remain prevalent, particularly in severe cases. This study aims to identify risk factors for early seizures in patients with TBI receiving prophylactic levetiracetam monotherapy and to develop a clinical scoring model for risk stratification. This study was conducted at an emergency medical center in Japan (2018–2022). In total, 168 patients with TBI were screened, and 147 severe cases met eligibility criteria. Exclusion criteria included age under 18, prehospital cardiac arrest, prior AED use, pregnancy, and alcohol dependency. All patients received prophylactic levetiracetam, with dosage and duration determined by neurocritical care physicians. The primary outcome was early seizures and associated risk factors. Secondary outcomes included hospital stay, functional outcomes, and in-hospital mortality. Multivariate logistic regression identified independent risk factors, and a clinical risk scoring model was developed. Early seizures occurred in 25 of the 147 patients. Multivariate analysis identified heart rate (odds ratio [OR]: 1.048, *p* < 0.001), creatinine level (OR: 1.535, *p* = 0.026), and acute subdural hematoma (OR: 5.861, *p* = 0.027) as independent risk factors. A clinical risk scoring model incorporating these variables demonstrated high predictive accuracy (area under the receiver operating characteristic curve: 0.828, 95% confidence interval: 0.725–0.931). Patients scoring >4.5 underwent electroencephalographic monitoring. This study presents a novel risk scoring model integrating heart rate, creatinine, and acute subdural hematoma to predict early seizures in patients with TBI despite prophylactic levetiracetam use. The model enables rapid risk stratification upon admission and may guide targeted neurocritical management.

## Introduction

Seizures occurring within the first week following traumatic brain injury (TBI) are generally defined as early post-traumatic seizures (EPTS).^1–4^ These seizures are distinguished from epileptic seizures and are classified as acute symptomatic seizures. EPTS is a well-recognized and severe complication of TBI, with an incidence ranging from 5% to 25%.^5–7^ However, in clinical practice, the incidence of EPTS varies widely depending on patient demographics, the mechanism of injury, and the severity of brain damage, with a notably higher frequency observed in patients with severe TBI.^8,9^ The underlying pathophysiology of EPTS is believed to involve a cascade of post-injury neurophysiological changes, including increased metabolic demand, intracranial hypertension, excessive neurotransmitter release, and abnormalities in cerebral perfusion.^10–14^ These factors contribute to secondary brain injury, potentially leading to neurological deterioration, increased mortality, and an elevated risk of progression to late post-traumatic epilepsy.^4,15,16^ Consequently, the prevention and management of EPTS in the acute phase of TBI are recognized as critical components of neurocritical care. Current clinical guidelines recommend the prophylactic administration of antiepileptic drugs (AEDs), particularly phenytoin or levetiracetam, for 7 days in patients with severe TBI or intracranial hemorrhage.^17,18^ Numerous studies have reported the efficacy of various AEDs in reducing the risk of EPTS.^3,19–23^ Additionally, several risk factors for EPTS have been identified, including intracranial hematoma, acute subdural hematoma, brain edema, younger age, prolonged loss of consciousness (>30 min), anterograde amnesia, focal neurological deficits, skull fracture, and penetrating injuries.^11,20–28^ However, despite prophylactic AED administration, a subset of patients still experiences EPTS, highlighting the limitations of current preventive strategies.^23,26,27^ The specific risk factors for the occurrence of seizures in these patients remain unclear, which poses challenges in clinical decision-making. In particular, monotherapy with a single AED may not be sufficient in some cases, and the possibility of nonconvulsive status epilepticus or subclinical seizure activity should not be overlooked. These considerations underscore the need for a more comprehensive approach, including enhanced risk stratification, long-term neurophysiological monitoring, and multimodal diagnostic strategies, in order to optimize the management of EPTS in patients with TBI.

## Objective

This study aimed to retrospectively analyze the cases of patients with TBI who were transported to our institution and received monotherapy with levetiracetam for early seizure prophylaxis. Patients were categorized according to the presence or absence of EPTS. Specifically, we examined initial vital signs, blood test results, and head computed tomography (CT) findings, comparing both groups to identify risk factors associated with the occurrence of seizures in patients who received monotherapy for seizure prophylaxis. The ultimate goal was to provide insights that may contribute to the acute-phase management of TBI.

## Methods and Participants

### Ethical considerations

This study was approved by the Ethics Committee of Nippon Medical School Hospital (O2022612) and conducted in accordance with its ethical standards and the Declaration of Helsinki (1975). Informed consent for the use of data collected during hospitalization was obtained from all patients under an opt-out consent policy for data protection. Since this study was a retrospective analysis of data collected during routine clinical practice, additional consent specifically for research participation was deemed unnecessary.

### Patient selection

This study included patients with TBI who were transported to our tertiary emergency medical center in Tokyo over a 5-year period from 2018 to 2022. Only cases classified as severe trauma based on Tokyo emergency medical service criteria were included. Patients under 18 years of age, those in cardiac arrest on arrival, those with pre-existing epilepsy or prior AED use, pregnant patients, and individuals with alcohol dependence were excluded. Only patients who received prophylactic AED therapy for early seizure prevention were included in the analysis. Upon admission, all patients underwent a thorough assessment, including vital sign evaluation, blood testing, and head CT imaging. Patients with TBI were managed by an intensive care unit (ICU) team that included neurointensivists. Continuous EEG monitoring was initiated at the discretion of the neurointensivists in cases where seizures were suspected or when patients exhibited unexplained neurological symptoms.

### Data collection

The collected data included demographic characteristics such as age and sex, as well as vital signs, including blood pressure, heart rate, and initial level of consciousness measured by the Glasgow Coma Scale. Arterial blood gas analysis was performed, including pH, lactate, and base excess levels. Laboratory findings included complete blood count and serum biochemical parameters. Head CT findings were also assessed, including the presence, size, and number of cerebral contusions, as well as the presence of subarachnoid hemorrhage, classified based on a thickness threshold of ≥3 mm. The presence of acute subdural hematomas was evaluated, including their thickness, categorized as ≥5 or ≥10 mm, and whether the hematoma was unilateral or bilateral. The presence of acute epidural hematomas, skull fractures, either single or multiple, and midline shift of ≥5 mm was also documented.

### Medication

The initiation of levetiracetam for early seizure prophylaxis was determined at the discretion of the neurointensivists, based on previously reported risk factors such as intracranial hematoma, acute subdural hematoma, brain edema, prolonged loss of consciousness (≥30 min), and focal neurological deficits. The dosage was adjusted as needed according to the clinical judgment of the attending physicians. According to the 2024 Pharmaceuticals and Medical Devices Agency prescribing information, levetiracetam was generally administered at a dose of 500 mg twice daily, infused intravenously over 15 min (total daily dose: 1,000 mg). Depending on the patient’s condition, the dose could be increased up to a maximum of 3,000 mg/day. Dose escalation was performed in increments of 1,000 mg/day at intervals of at least 2 weeks.

### Definition of early seizures

Patients were classified as experiencing EPTS if they met either of the following criteria: (1) documentation of epileptic activity in the medical records or nursing flow sheets, leading to the administration of AEDs, including benzodiazepines, an increased dose of levetiracetam, or the addition of another AED; or (2) EEG-confirmed seizure activity, including suspected nonconvulsive status epilepticus, diagnosed by neurointensivists.

### Primary and secondary outcomes

The primary outcome was the incidence of EPTS and the identification of associated risk factors. Secondary outcomes included the length of hospital stay, clinical outcomes, and in-hospital mortality.

### Statistical analysis

Continuous variables were expressed as medians with interquartile ranges, while categorical variables were reported as frequencies and percentages. Fisher’s exact test was used to compare categorical variables, and the Wilcoxon rank-sum test was applied for continuous variables. To determine the association between patient characteristics and early seizure occurrence, variables with *p* values of <0.05 in a univariate analysis and those deemed clinically relevant were included in the multivariate logistic regression model. Variables that remained significant in the multivariate model were incorporated into a scoring system, with regression coefficients used to assign point values to each parameter. The relationship between the risk score and seizure probability was assessed to establish a clinically applicable risk stratification model. The diagnostic accuracy of the scoring system was evaluated using receiver operating characteristic curve analysis, and the area under the curve was calculated. The Youden index was used to determine the optimal cutoff value, achieving maximum sensitivity and specificity. All statistical tests were two-tailed, with statistical significance set at 0.05. Analyses were conducted using StatFlex Plus Ver. 7 (View Flex, Japan).

## Results

**FIG. 1.**
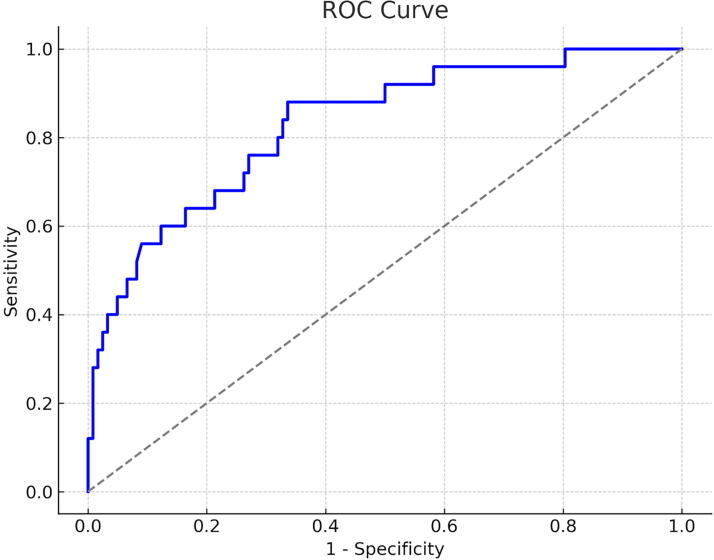
Receiver operating characteristic (ROC) curve of the score model for the derivation and validation cohorts. Area under the curve, 0.828; sensitivity, 0.880; specificity, 0.664. The cutoff value was 0.118.

A total of 168 patients were screened for inclusion in the study. None of the patients transported to our institution had a history of epilepsy, prior AED use, alcohol dependence, pregnancy, or chronic renal failure requiring maintenance dialysis. After excluding 13 patients in cardiac arrest on arrival and 8 patients <18 years of age, a total of 147 patients were included in the final analysis. There were 27 patients with multiple trauma, defined as having an Abbreviated Injury Scale (AIS) score of ≥3 in two or more regions; however, none of these patients presented with signs of shock on arrival. On arrival, all patients underwent a parameter assessment, and all cases involved blunt trauma. Among the included patients, 25 experienced EPTS, while 122 did not. A comparison between the seizure and non-seizure groups revealed no significant differences in age, sex, head AIS score, Injury Severity Score (ISS), or Acute Physiology and Chronic Health Evaluation II (APACHE II) score. Univariate regression analysis identified initial heart rate, hematocrit level, and creatinine level as factors associated with early seizure occurrence. In addition, head CT was performed for all patients immediately after arrival, assessing the presence, size, location, and number of cerebral contusions, as well as the presence, thickness, location, and number of acute subdural hematomas; acute epidural hematomas and their thickness; traumatic subarachnoid hemorrhage; and skull fractures, either single or multiple. Univariate analysis of these imaging findings revealed that the presence of acute subdural hematoma was the only variable significantly associated with EPTS (Table 1). For the secondary outcomes, comparison of various parameters between the seizure and non-seizure groups revealed that the proportion of patients with favorable outcomes was significantly lower in the seizure group. Additionally, the duration of mechanical ventilation was significantly longer among patients who experienced seizures. Although there was no significant difference in mortality between the two groups, a tendency for prolonged hospitalization was observed in the seizure group (Table 2). To evaluate risk factors for seizure occurrence, a logistic regression analysis using Firth’s penalized likelihood method was performed. This method is particularly useful in reducing small-sample bias when event counts are low. In this analysis, seizure occurrence was set as the dependent variable, while heart rate, creatinine level, and presence of acute subdural hematoma were included as independent variables. The results of the multivariate analysis showed that early seizure occurrence was significantly associated with an increase in heart rate (odds ratio [OR]: 1.048; 95% confidence interval [CI]: 1.024–1.073; *p* < 0.001), an increase in creatinine level (OR: 1.535; 95% CI: 1.053–2.239; *p* = 0.026), and the presence of an acute subdural hematoma (OR: 5.861; 95% CI: 1.219–28.172; *p* = 0.027) (Table 3). To evaluate the predictive performance of the multivariate logistic regression model, a receiver operating characteristic curve analysis was performed (Fig. 1). The c-index, or area under the curve, was 0.828 (95% CI: 0.725–0.931), indicating strong discriminatory ability. The optimal cutoff value, as determined using the Youden index, which identifies the point that maximizes the sum of sensitivity and specificity, was 0.112, with sensitivity of 0.880 and specificity of 0.664. These results suggest that this model is effective in predicting seizure occurrence in patients on prophylactic AED therapy. The major explanatory variables were categorized to further enhance the clinical utility of this predictive model. Heart rate and creatinine level were treated as continuous variables, while the presence of an acute subdural hematoma was classified as present (1) or absent (0). A scoring system was developed based on the coefficients from the multivariate logistic regression model. The risk score was calculated using the following equation:

Score = (Heart rate × 0.02) + (Creatinine × 0.2) + (Acute subdural hematoma × 2).

**Table 1.** Univariate Regression Analysis of Factors Associated with Early Post-Traumatic Seizures

Parameter	Odds ratio	95% CI	*p* Value
Age, years	1.014	0.994–1.037	0.189
Male	1.673	0.566–5.951	0.400
AIS (head)	1.625	0.917–2.879	0.092
ISS	0.984	0.941–1.029	0.476
Levetiracetam	1.459	0.805–2.553	0.194
Operation	1.687	0.695–4.409	0.261
GCS score	1.161	0.674–2.054	0.595
Blood pressure, mmHg			
Systolic	1.008	0.995–1.022	0.224
Diastolic	1.001	0.980–1.023	0.905
Heart rate, bpm	1.026	1.006–1.047	0.001
pH	0.898	0.042–46.786	0.950
PaCO_2_, mmHg	0.995	0.954–1.009	0.701
ABE, mmol/L	0.989	0.924–1.072	0.763
Glu, mg/dL	1.001	0.993–1.008	0.773
Lac, mmol/L	1.000	0.985–1.012	0.952
WBC	0.14	0.05–0.32	<0.001
Hb	1.95	1.36–2.80	<0.001
Plt	1.24	1.10–1.47	0.003
BUN, mg/dL	1.022	0.987–1.054	0.190
Cre, mg/dL	1.229	1.012–1.500	0.002
Alb, g/dL	0.751	0.342–1.687	0.478
CRP, mg/dL	1.101	0.906–1.301	0.277
D-dimer, mg/L	0.993	0.981–1.002	0.211
SAH	0.610	0.238–1.567	0.305
Contusion (single)	1.273	0.537–3.082	0.585
Contusion (≥10 mm)	1.100	0.655–1.783	0.706
Contusion (multiple)	0.767	0.239–2.087	0.624
Contusion (location)	1.078	0.415–2.388	0.863
Contusion (bilateral)	2.263	0.832–5.829	0.096
SDH	5.610	1.553–36.043	0.00
SDH (≥10 mm)	1.925	1.190–3.113	0.007
SDH (falx)	2.889	1.043–7.643	0.035
SDH (bilateral)	3.000	1.020–8.313	0.038
EDH	0.620	0.138–1.997	0.468
EDH (≥10 mm)	0.798	0.302–1.586	0.575
Skull fracture	0.802	0.250–2.187	0.684
Midline shift (≥5 mm)	2.034	0.828–4.918	0.115

ABE, actual base excess; AIS, Abbreviated Injury Scale; Alb, albumin; bpm, beats per minute; BUN, blood urea nitrogen; CI, confidence interval; Cre, creatinine; CRP, C-reactive protein; EDH, epidural hematoma;  EPS, early post traumatic seizure; GCS, Glasgow Coma Scale; Glu, glucose; Hb, hemoglobin; ISS, Injury Severity Score; Lac, lactate; PaCO_2_, arterial partial pressure of carbon dioxide; Plt, platelets; SAH, subarachnoid hemorrhage; SDH, subdural hematoma; WBC, white blood cell count.

**Table 2.** Comparison of Secondary Outcomes Between Seizure and Non-Seizure Groups

Parameter	EPS +	EPS −	*p* Value
Cases, *n*	25	122	
Age, years	75 (43–72)	67 (27–57)	0.209
Male, *n*	93 (76%)	21 (84%)	0.766
GCS score	9 (6–13.3)	9 (6–13)	0.624
Operation, *n*	8 (32%)	54 (44%)	0.457
Favorable outcome, *n*	7 (28%)	82 (67%)	0.047
Death, *n*	1 (4%)	9 (7%)	0.564
Levetiracetam (dose)	1,000 (1,000–2,000)	1,000 (1,000–1,000)	0.150
AIS (head)	5 (4–5)	4 (4–5)	0.102
ISS	25 (16–25)	25 (16–29)	0.345
Length of hospital stay, days	22 (15.8–37.5)	18.5 (10.0–29.0)	0.061
Dependent on ventilation	17.0 (9.3–26.8)	8.5 (3.0–18.0)	0.015
Blood pressure, mmHg	155 (127–173.3)	165 (125–191)	0.317
Heart rate, bpm	105 (89.8–127)	85 (74–100)	<0.001
BUN, mg/dL	15.2 (10.8–22.4)	17.0 (12.7–20.2)	0.337
Cre, mg/dL	0.96 (0.65–1.24)	0.82 (0.67–0.98)	0.177
Alb, g/dL	3.9 (3.4–4.2)	3.9 (3.5–4.3)	0.567
CRP, mg/dL	0.1 (0.0–0.9)	0.1 (0.0–0.3)	0.321
D-dimer, mg/L	12.7 (8.0–38.5)	28.4 (11.6–61.4)	0.075
SAH, *n*	17 (68%)	94 (77%)	0.716
SDH, *n*	23 (92%)	82 (67%)	0.329
EDH, *n*	3 (12%)	22 (18%)	0.531
Contusion, *n*	14 (56%)	61 (50%)	0.759
Skull fracture, *n*	5 (20%)	29 (24%)	0.745
Midline shift, *n*	11 (44%)	34 (28%)	0.263

AIS, Abbreviated Injury Scale; Alb, albumin; bpm, beats per minute; BUN, blood urea nitrogen; Cre, creatinine; CRP, C-reactive protein; EDH, epidural hematoma; GCS, Glasgow Coma Scale; ISS, Injury Severity Score; SAH, subarachnoid hemorrhage; SDH, subdural hematoma.

**Table 3.** Multivariate Logistic Regression Analysis of Factors Associated with Early Traumatic Epilepsy

Parameter	Odds ratio	95% CI	*p* Value
HR	1.048	1.024–1.073	<0.001
Cre	1.535	1.053–2.239	0.026
SDH	5.861	1.219–28.172	0.027

CI, confidence interval; Cre, creatinine; HR, heart rate; SDH, subdural hematoma.

**FIG. 2.**
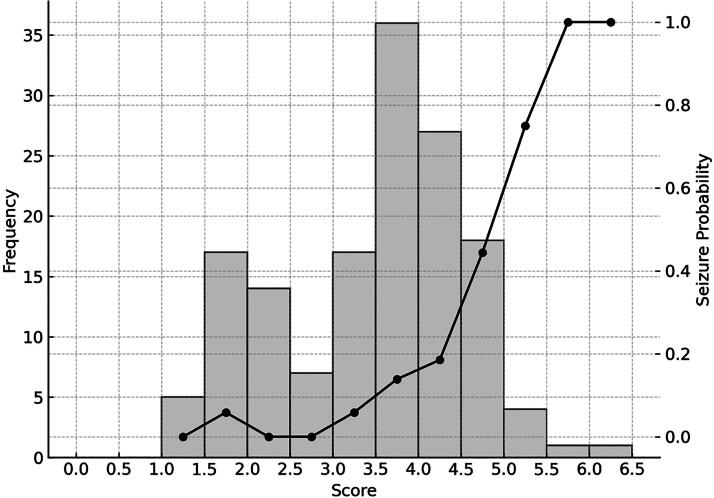
Score distribution and early seizure probability. The left vertical axis indicates the number of cases, and the right vertical axis indicates the probability of early seizure occurrence. The estimated probability of early post-traumatic epilepsy in patients with traumatic brain injury is calculated as Score = (HR × 0.02) + (Cre × 0.20) + (SDH × 2.0), where SDH is the subdural hematoma (absent, 0; present, 1), HR is heart rate (beats per min, continuous), and Cre is creatinine level (mg/dL, continuous).

This model enabled the calculation of a risk score for each patient based on individual characteristics, facilitating risk stratification for seizure occurrence. The results indicated that higher scores were associated with an increased risk of poor clinical outcomes (Fig. 2).

### ICU management

All patients included in the study were admitted to the ICU and managed according to the latest clinical guidelines. Cases requiring immediate surgical intervention underwent emergency procedures following initial assessment. Additionally, patients who required surgery within the first 7 days of hospitalization also underwent emergency operations. The initial dose of levetiracetam was administered within 6 h in all cases. Intravenous administration was used during the initial phase, and oral administration was continued in patients who could tolerate enteral intake. Serum drug levels were not monitored during the study period.

## Discussion

In this study, we investigated the risk factors for EPTS in 25 of the 147 patients who received guideline-based prophylactic AED therapy in the acute phase of TBI. Despite prophylactic AED administration, a certain proportion of patients still experience EPTS, which remains a clinical challenge.^4,29,30^ Prompt identification and management of such cases are crucial for improving clinical outcomes.^31–33^ Therefore, we developed a simple yet accurate clinical scoring model to predict EPTS in patients with TBI who were receiving prophylactic AED therapy. Scoring systems have been widely utilized in various clinical settings.^34–36^ Our model is based on three readily assessable clinical factors: heart rate at admission, creatinine level, and the presence of acute subdural hematoma. This enables rapid risk stratification soon after arrival. The area under the curve was 0.828, with sensitivity of 0.880 and specificity of 0.664, demonstrating high discriminative ability. The cutoff value was 0.118 (95% CI: 0.725–0.931), confirming its practicality and reliability as a predictive tool.

Elevated serum creatinine levels on admission were significantly associated with increased risk of EPTS. This may reflect broader neurophysiological disruptions linked to renal dysfunction. Acute or chronic kidney impairment can exacerbate neurotoxicity through accumulation of uremic toxins, oxidative stress, and systemic inflammation, which collectively lower the seizure threshold and promote cortical hyperexcitability.^37^ Furthermore, compromised renal function may result in disruption of the blood–brain barrier, facilitating the translocation of neurotoxic metabolites.^38^ Levetiracetam, which is predominantly excreted unchanged via the kidneys, may accumulate under conditions of renal insufficiency. Although it has a wide therapeutic index, paradoxical seizure exacerbation due to elevated drug levels has been documented in the context of renal impairment.^39^ Conversely, augmented renal clearance may lead to subtherapeutic AED levels, although this was not a major issue in our cohort. Creatinine level may thus not only serve as a renal biomarker but also potentially reflect heightened cortical excitability in the acute phase of TBI.

Tachycardia at admission emerged as an independent predictor of EPTS. Following severe TBI, autonomic dysregulation—particularly sympathetic hyperactivity—can trigger paroxysmal elevations in catecholamines, blood pressure fluctuations, and altered heart rate variability.^40^ Such dysregulation, known as paroxysmal sympathetic hyperactivity, is not only a marker of central autonomic imbalance but may also exacerbate neuronal excitability and predispose to seizure onset. Moreover, recent evidence suggests that pre-ictal increases in heart rate may reflect imminent seizure activity, supporting the hypothesis that autonomic markers are intricately linked to cortical instability.^41^

Acute subdural hematoma remains among the most epileptogenic lesions in the context of TBI. Cortical irritation from the hematoma, impaired perfusion, and the release of iron and hemoglobin breakdown products promote oxidative injury and excitotoxicity, fostering epileptogenesis.^42^ Additionally, microglial activation and reactive gliosis during hematoma resorption may contribute to sustained network instability and seizure susceptibility. Clinically, acute subdural hematoma has consistently been reported as a leading risk factor for EPTS.^43^ The present findings reinforce this notion, underscoring the critical need for vigilant monitoring and targeted interventions in patients with acute subdural hematoma.

Collectively, elevated creatinine, tachycardia, and acute subdural hematoma appear to converge on a final common pathway of increased neuronal excitability, albeit via distinct mechanistic routes: systemic metabolic stress, autonomic dysregulation, and focal structural insult, respectively. These factors, individually and synergistically, potentiate early seizure risk in the vulnerable brain. The clinical scoring system developed herein captures these dimensions in a parsimonious yet robust manner, facilitating rapid bedside risk stratification in severe TBI. Given the persistent incidence of EPTS even under guideline-based prophylaxis, such a tool may aid in refining neurocritical care protocols and prioritizing candidates for intensive electroencephalographic surveillance. Further validation in multicenter prospective cohort studies is warranted to establish the model’s generalizability and clinical utility. Our findings suggest that patients with a score greater than 4.5 have a significantly increased risk of experiencing seizures, even under prophylactic AED therapy. These patients may require not only inpatient observation but also ICU management and more rigorous neurocritical care interventions, including electroencephalographic monitoring. Early identification and intervention for EPTS could serve as a crucial step toward improving patient outcomes. Previous studies have reported EPTS as an independent predictor of a poor prognosis,^44–47^ and our study also demonstrated that the occurrence of seizures was associated with a lower rate of favorable outcomes, prolonged mechanical ventilation, and extended hospital stays. These findings emphasize the importance of effective seizure prevention in patients with TBI. Levetiracetam was used as the first-line AED in all cases, due to its efficacy and safety, and because—unlike phenytoin—it does not require therapeutic drug monitoring.^4,5,16,24,48,49^ While different AEDs might have yielded different results, the widespread use of levetiracetam in clinical practice supports its appropriateness as a first-line therapy.^50–52^ The dosage used in this study was within the approved range in Japan, and given that no patients with renal failure were included, the dosing was considered appropriate.^53–55^ Additionally, the risk of EPTS varies depending on the severity of TBI.^4^ In our cohort, which predominantly consisted of severe TBI cases, the seizure incidence remained high at 17.7% despite prophylactic treatment. This suggests that our scoring system may be particularly valuable in level 1 trauma centers, where cases of severe TBI are frequently encountered. However, its applicability to mild TBI cases remains uncertain and warrants further investigation.

## Limitations

This study had several limitations. First, as a single-center retrospective observational study, the external validity of the findings is limited. Since all data were collected from a single institution, regional variations in disease prevalence and differences in treatment approaches among medical facilities were not accounted for. Consequently, the results may not be entirely generalizable to other TBI populations. Second, the study included a relatively small number of patients with TBI who were admitted to the ICU, which may have affected the statistical power of the analysis. Additionally, because our institution is a tertiary emergency medical center, the severity of cases transported was relatively high. This could have influenced the observed relationship between brain injury and poor outcomes, imposing certain constraints on the conclusions drawn. Third, levetiracetam was the only agent used for seizure prophylaxis in all cases, and the study did not evaluate the efficacy of alternative AEDs such as phenytoin. To address these limitations, future research should include multicenter collaborative studies and prospective data collection to ensure greater generalizability. Furthermore, validation of this predictive model through randomized controlled trials is necessary to establish its clinical utility and external validity.

## Conclusion

In this study, we developed and evaluated a scoring model for assessing the risk of EPTS in the acute phase of TBI. We identified heart rate at admission, creatinine level, and the presence of acute subdural hematoma as significant predictors of EPTS, even under prophylactic AED therapy. Our scoring system enables early risk stratification at the initial phase of transport, guiding appropriate management strategies. Further multicenter studies are required for validation of external applicability.

## Data Availability

The data that support the findings of this study are available on request from the corresponding author. The data are not publicly available due to privacy or ethical restrictions.
